# Knowledge, Attitude, and Practice (KAP) Survey on the Management of Multidrug-Resistant Gram-Negative Infections With Innovative Antibiotics: Focus on Ceftazidime–Avibactam

**DOI:** 10.7759/cureus.39245

**Published:** 2023-05-19

**Authors:** Abhisek Routray, Akshata Mane

**Affiliations:** 1 Medical Affairs, Pfizer India, Mumbai, IND

**Keywords:** ceftazidime avibactam, enterobacterales, rapid diagnosis, multidrug-resistant bacteria, oxa-48

## Abstract

Background: Antimicrobial resistance (AMR) is a major public health dilemma and a chief health concern globally. The rising incidence of resistance against carbapenems, which are considered most effective against gram-negative bacteria, has added to the concern and has limited the number of available treatment options. Newer antibiotic options may be required to tackle the mounting concern of antibiotic resistance. However, only a few antimicrobials are in the pipeline for managing infections instigated by multidrug-resistant (MDR) gram-negative bacteria. This justifies the prudent application of already available antibiotics. Among newer antibiotics available to healthcare professionals (HCPs), ceftazidime-avibactam (CAZ-AVI) has shown good efficacy in the management of MDR gram-negative infections.

Method: A cross-sectional survey on the knowledge, attitude, and practices (KAP) among HCPs was carried out using a questionnaire comprising 21 parameters related to AMR patterns on the need for innovative antibiotics to manage MDR gram-negative infections and the usage of CAZ-AVI by HCPs while managing such infections. The KAP scores were calculated to rank respondents' KAP levels.

Result: Out of the 204 study respondents, the majority (~80%) (n=160) believed that renewed efforts should be made to seek antimicrobial agents that will add to the armamentarium of treatment options for MDR gram-negative infections. CAZ-AVI is an important treatment alternative for managing MDR gram-negative infections (n=90, 45%). Further, it can be the first choice of definitive therapy for oxacillinases (OXA)-48-producing carbapenem-resistant *Enterobacterales *(n=84, 42%). HCPs also believed that the use of CAZ-AVI in clinical practice will require high levels of antimicrobial stewardship (n=100, 49%).

Conclusion: Novel and innovative antibiotics are the need of the hour in the management of MDR gram-negative infections. CAZ-AVI has established its effectiveness in treating these infections; however, the molecule must be utilized prudently while keeping stewardship principles in mind.

## Introduction

Antimicrobial resistance (AMR) poses a substantial public health concern globally. Antimicrobials should be used judiciously to slow AMR and improve patient outcomes. Antibiotic-resistant bacteria are rapidly emerging due to the lack of newer antibiotics and the unreasonable prescription of existing antibiotics. This is detrimental to the effective and economical management of gram-negative bacterial infections globally [1,2]. The increasing prevalence of multidrug-resistant (MDR) gram-negative bacteria, such as *Enterobacterales* (especially *Klebsiella pneumoniae *and *Escherichia coli*), *Pseudomonas aeruginosa*, and *Acinetobacter baumannii*, is connected to growing morbidity, mortality, and hospital stay [3,4]. With the increasing resistance of certain bacterial strains to multiple drugs, resulting in difficult-to-treat resistant infections, choosing empiric antimicrobial therapy has become challenging for healthcare professionals (HCPs) to care for very ill hospitalized patients [5,6]. The clinical challenges generated by carbapenem-resistant gram-negative bacteria have rekindled interest in finding novel ways to treat these illnesses. Newer antibiotics, especially those that have shown significant clinical benefits and those targeting carbapenem-resistant *Enterobacterales* (CRE) and carbapenem-resistant *P. aeruginosa *avibactam, such as meropenem-vaborbactam, ceftolozane-tazobactam, plazomicin, eravacycline, omadacycline, imipenem-cilastatin-relebactam, cefiderocol, and fourth-generation fluoroquinolones (delafloxacin), are few such molecules that have strengthened the clinician’s armamentarium [6-8].

Ceftazidime-avibactam (CAZ-AVI), a combination of ceftazidime and novel β-lactamase inhibitor avibactam that is effective against class A, class C, and class D β-lactamases, such as extended-spectrum beta-lactamases, AmpC β-lactamases (AmpC), and *K. pneumoniae* carbapenemase (KPC) enzymes, and oxacillinases (OXA)-48 carbapenemases have proven to be instrumental in the management of infections caused by pathogens that produce these enzymes. The combination is effective against a variety of MDR gram-negative bacteria, especially in hospitalized patients with CRE and difficult-to-treat *P. aeruginosa* strains [9-11]. However, studies conducted in hospital settings have shown that the use of innovative molecules like CAZ-AVI needs good antimicrobial stewardship (AMS) to ensure the longevity of the molecule, owing to the dry pipeline [12-14].

Currently, AMS is one of the three components of a comprehensive plan to strengthen health systems [15]. Studies have demonstrated that AMS programs should not be vertical, rather they should be implemented across health systems, assisting medical professionals to ensure that each patient receives the most effective antibiotic at the precise dosage for the correct amount of time and not as a last resort. Hence, timely implementation of AMS at healthcare facilities reduces the need for and expense of antibiotics while also reducing the frequency of illnesses related to medical care [14,15].

To understand the current medical approach toward the treatment of MDR gram-negative infections with innovative antibiotics, such as CAZ-AVI, an online KAP survey was administered among Indian HCPs. This article aims at comprehending the current scenario of MDR gram-negative infections in India, the unmet needs with regard to MDR gram-negative infections prevalent in the country, and the role of innovative antibiotics, such as CAZ-AVI, when treating MDR gram-negative infections.

## Materials and methods

Study design and setting

This cross-sectional study (KAP survey) was conducted among HCPs in India. The study comprised 204 HCPs (infectious disease [ID] specialists, pulmonologists, intensivists, gastroenterologists, nephrologists, and microbiologists) from across the country. The sample size was based on the number of HCPs who responded to all the questions in the survey. This survey was carried out from July 2022 to August 2022 through an online questionnaire, which was designed to gather HCPs’ perspectives. The 21-point KAP survey focused on the assessment of HCPs on the role of innovative antibiotics in tackling the unmet needs in managing multidrug resistance in gram-negative bacteria. The questions were broadly classified as information about the respondents and their KAP with regard to the treatment of MDR gram-negative infections. The identity of participants was not requested as a part of the questionnaire, and their answers were kept confidential.

Data collection

The questionnaire had both closed- and open-ended questions. The majority of the queries were based on a five-point Likert scale. Other domains assessed were HCPs’ professional characteristics, their practical knowledge, and their approach toward the use of antibiotics in their everyday practice. This mixed approach was devised to obtain a holistic view of the opinions of the HCPs. The anonymity of the participants was maintained. A sample of the questionnaire has been provided in Table 1.

**Table 1:** KAP survey questionnaire for HCPs

Professional characteristics of the respondents (N=204)							
Category	Variables						
Please select your primary specialty	ID specialist	Intensivist	Pulmonologist	Nephrologist	Surgeon	Gastroenterologist	Microbiologist
Number of years of experience	<5 years		5–10 years		10–20 years		>20 years
KAP survey questionnaire							
Questions			Responses				
The global shortage of innovative antibiotics fuels the emergence and spread of drug resistance.			Strongly disagree	Disagree	Neither agree nor disagree	Agree	Strongly agree
The resistance problem demands that a renewed effort should be made to seek antibacterial agents effective against pathogenic bacteria resistant to current antibiotics.			Strongly disagree	Disagree	Neither agree nor disagree	Agree	Strongly agree
Newer antibiotics will add to the armamentarium of treatment options for MDR gram-negative infections and help tackle AMR.			Strongly disagree	Disagree	Neither agree nor disagree	Agree	Strongly agree
CAZ–AVI is an important tool in the armamentarium for managing MDR gram-negative infections.			Strongly disagree	Disagree	Neither agree nor disagree	Agree	Strongly agree
In view of the limited availability of treatment options, infections with carbapenem-resistant organisms are associated with higher mortality rates.			Strongly disagree	Disagree	Neither agree nor disagree	Agree	Strongly agree
CAZ–AVI can be the first choice of definitive therapy for OXA-48-producing CRE			Strongly disagree	Disagree	Neither agree nor disagree	Agree	Strongly agree
Use of CAZ–AVI in clinical practice requires high levels of AMS.			Strongly disagree	Disagree	Neither agree nor disagree	Agree	Strongly agree
One of the major challenges of using CAZ–AVI is the lack of a rapid diagnostic tool.			Strongly disagree	Disagree	Neither agree nor disagree	Agree	Strongly agree
In my experience, early and appropriate use of CAZ–AVI leads to better patient outcomes when susceptible.			Strongly disagree	Disagree	Neither agree nor disagree	Agree	Strongly agree
Which are the most common gram-negative nosocomial pathogens you encounter in your ICU? (Select multiple options, if applicable.)				Escherichia coli	Klebsiella pneumoniae	Pseudomonas aeruginosa	Acinetobacter baumannii
Which are the most common nosocomial infections you encounter in your ICU? (Select multiple options, if applicable.)				Sepsis	Hospital-acquired pneumonia	Urinary tract infection	Intra-abdominal infection
To detect a common molecular mechanism of carbapenem resistance in *Enterobacterales*, such as OXA-48 and NDM-1, in my healthcare setting.					I order a rapid diagnostic molecular test	I do not order a rapid diagnostic test	A molecular testing facility is not available at the facility
Commonly used methods to detect the susceptibility of CAZ–AVI in my hospital setting. (Select multiple options, if applicable.)			Carba R	Vitek^®^-2	E test	Disk diffusion	All of the above
I use CAZ–AVI in the following while treating MDR gram-negative infections. (Select multiple options, if applicable.)			When local epidemiology is known, and the organism is carbapenem-resistant	When the organism is susceptible to CAZ–AVI	Based on rapid diagnostic test results	As a polymyxin sparer	As a carbapenem sparer
CAZ–AVI is considered a treatment option for the following infections in my hospital setting. (Select multiple options, if applicable.)			Hospital-acquired pneumonia	Intra-abdominal infection	Complicated urinary tract infection	Bacteremia	Hospital-acquired pneumonia
How early is CAZ–AVI used for the management of MDR gram-negative infections in your hospital setting?			<24 hours	<48 hours	<72 hours	<5 days	
AMR: Antimicrobial resistance; CAZ–AVI: Ceftazidime–avibactam; CRE: Carbapenem-resistant *Enterobacterales*; ICU: Intensive care unit; ID: Infectious disease; KAP: Knowledge, attitude, and practice; MDR: Multidrug-resistant; NDM-1: New Delhi metallo-beta-lactamase 1; OXA-48: Oxacillinase 48.							

Statistical analysis

The data was analyzed using Microsoft Excel (Microsoft, Washington, United States). Descriptive analysis was used for the results obtained to sort the responses under categories and to determine the percentages of survey participants under each of them.

## Results

Professional features of the respondents

**Figure 1:**
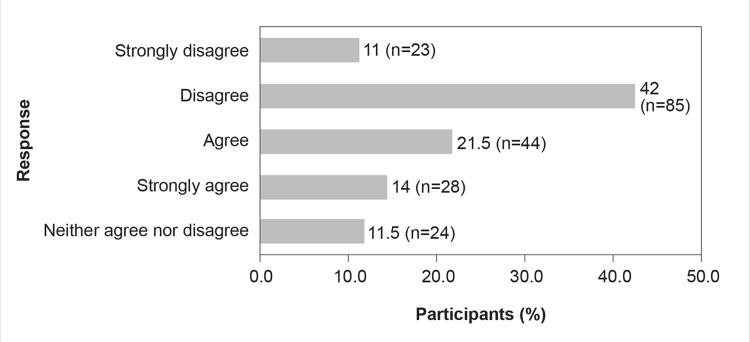
Global shortage of innovative antibiotics fuels the emergence and spread of drug resistance

**Figure 2:**
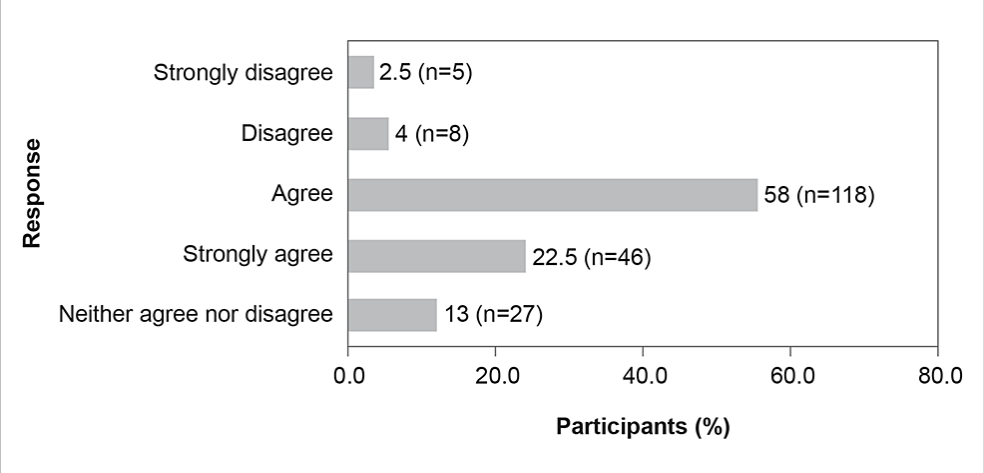
The resistance problem demands that a renewed effort should be made to seek antibacterial agents effective against pathogenic bacteria resistant to current antibiotics

**Figure 3:**
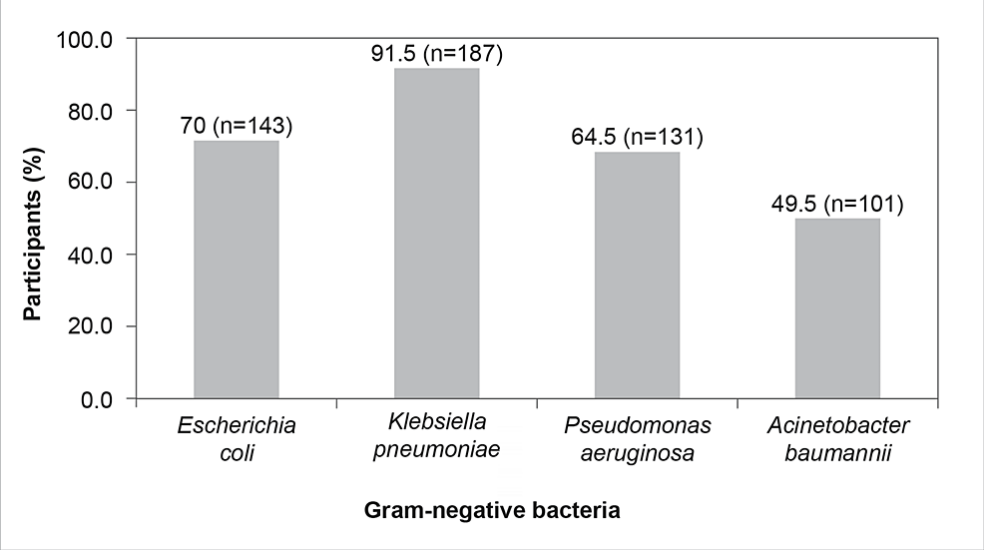
Which are the most common gram-negative nosocomial pathogens you encounter in your ICU? (Select multiple options, if applicable)

**Figure 4:**
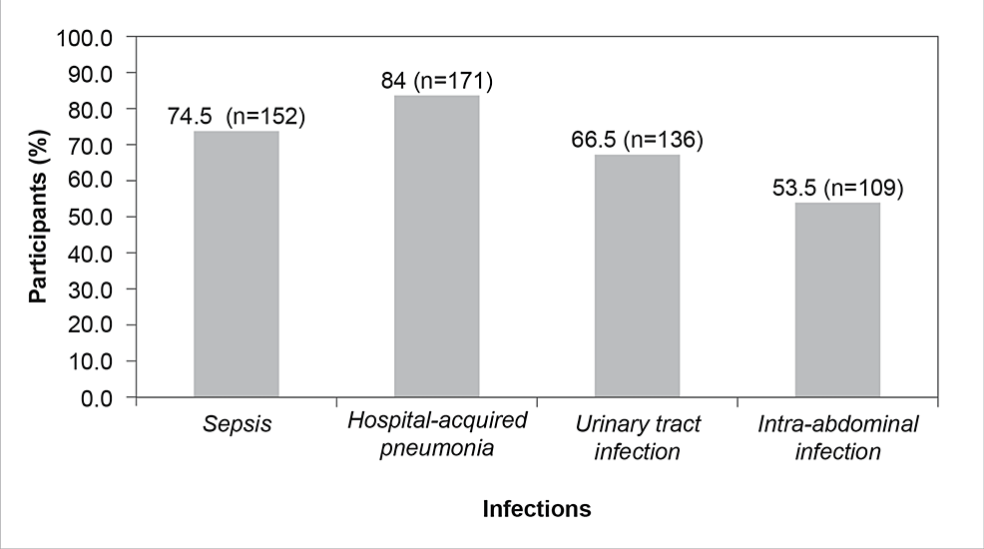
Which are the most common nosocomial infections encountered in your ICU? (Select multiple options, if applicable)

**Figure 5:**
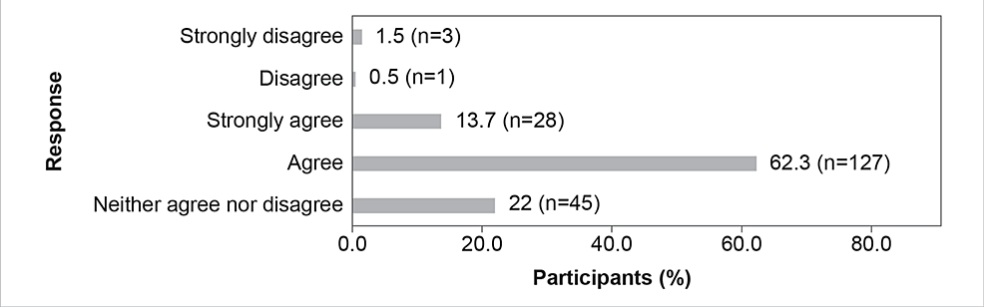
Newer antibiotics will add to the armamentarium of treatment options for MDR gram-negative infections and help tackle AMR

The participants’ responses to nosocomial infections in Indian hospital settings and the worldwide shortage of advanced antibiotics are illustrated in Figures 1-5. The majority of the participants (n=108, 53%) felt that the global shortage of innovative antibiotics does not fuel the appearance and spread of drug resistance (Figure 1).

Further, about 80.5% (n=164) of the participants believed that efforts should be made to maintain the effectiveness of antimicrobial agents against pathogenic bacteria resilient to current antibiotics (Figure 2).

Role of CAZ-AVI in managing MDR gram-negative infections

According to the participants, the most common gram-negative nosocomial pathogens encountered in Indian ICUs were *K. pneumoniae* (n=187, 91.5%), followed by *E. coli* (n=143, 70%), *P. aeruginosa* (n=134, 64.5%), and *A. baumannii* (n=101, 49.5%) (Figure 3).

Based on the survey results, hospital-acquired pneumonia (n=171, 84%) was the most encountered nosocomial infection, followed by sepsis (n=152, 74.5%), urinary tract infection (n=136, 66.5%), and intra-abdominal infection (n=109, 53.5%) (Figure 4).

The majority of the participants (n=155, 76%) agreed that novel antibiotics will add to the already existing armamentarium of treatment options for MDR gram-negative bacterial infections (Figure 5).

Use of CAZ-AVI in Indian hospital settings

**Figure 6:**
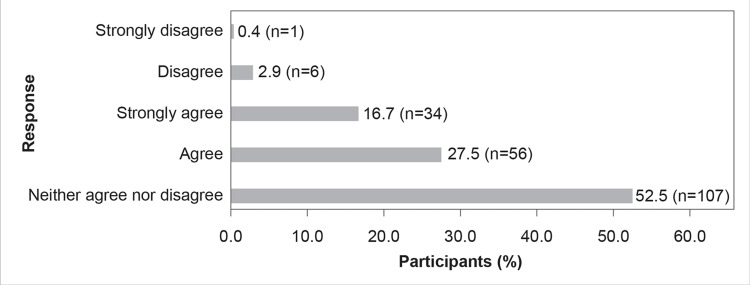
CAZ-AVI is an important tool in the armamentarium for managing MDR gram-negative infection

**Figure 7:**
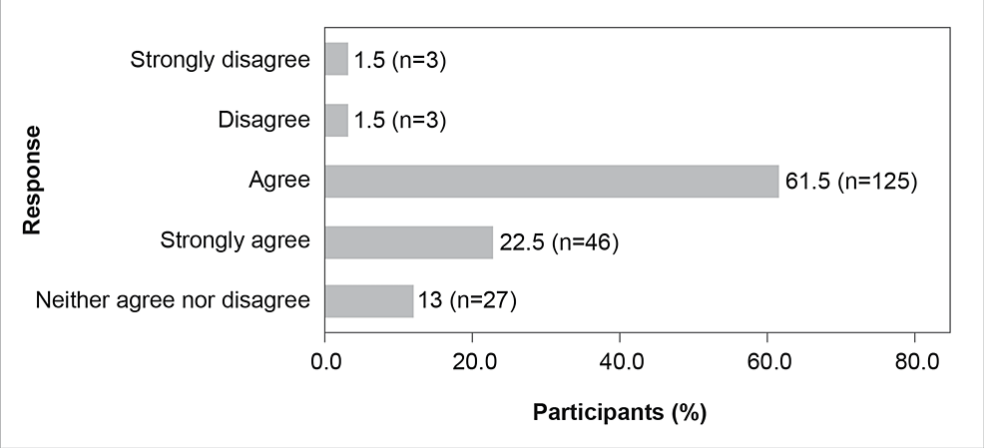
In view of the limited availability of treatment options, infections with carbapenem-resistant organisms are associated with higher mortality rates

**Figure 8:**
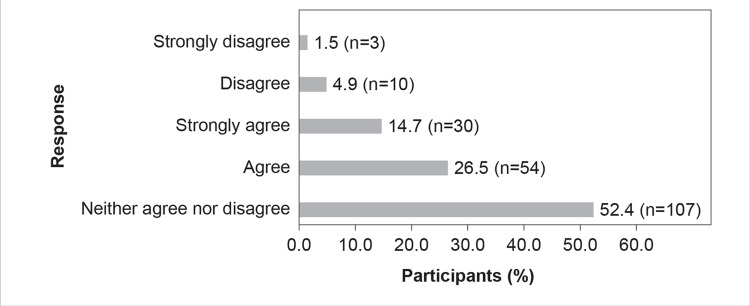
CAZ-AVI can be the first choice of definitive therapy for OXA-48-producing CRE

**Figure 9:**
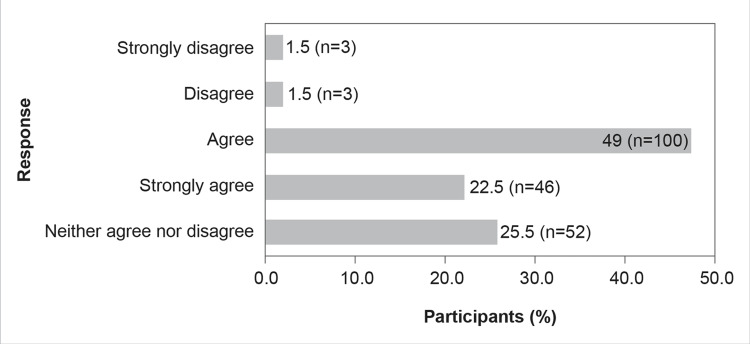
Use of CAZ-AVI in clinical practice requires high levels of AMS

**Figure 10:**
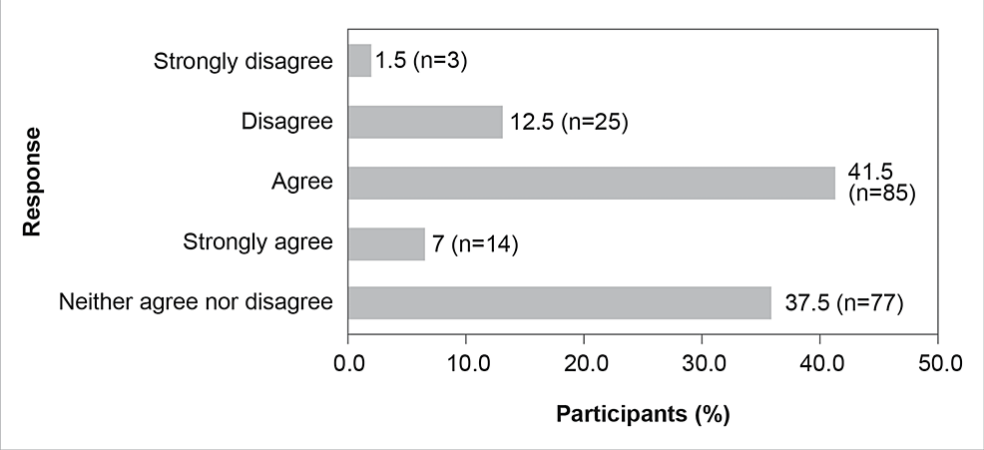
One of the major challenges of using CAZ-AVI is the lack of a rapid diagnostic tool

Responses of the HCPs to the questions on the role of CAZ-AVI in managing MDR gram-negative infections are demonstrated in Figures 6-10. Figure 6 illustrates the importance of CAZ-AVI in the armamentarium for managing MDR gram-negative infections.

The majority (84%, n=171) of the participants felt that infections due to carbapenem-resistant organisms were associated with a high mortality rate due to the limited options available for treating these infections (Figure 7).

Although 52.5% (n=107) of the study respondents were unsure on whether CAZ-AVI is an important armamentarium in Indian hospital settings for managing MDR gram-negative infections (opting for neither agree nor disagree), a good number (44.2%, n=90) of respondents agreed that CAZ-AVI was indeed essential in managing MDR gram-negative infections. Nearly 41.2% (n=84) of participants in the present KAP survey agreed that CAZ-AVI could be the first choice of definitive therapy for OXA-48-producing CRE, whereas only 6.4% (n=13) disagreed (Figure 8).

Although results reflected that CAZ-AVI was an effective solution to the rising MDR-associated gram-negative organisms, almost half of the participants (49%, n=100) agreed that the use of CAZ-AVI necessitates a high level of AMS (Figure 9).

Overall, 48.5% (n=99) of the participants agreed that one of the major challenges of using CAZ-AVI was the lack of rapid diagnostic tools (Figure 10). Probably, leveraging additional rapid diagnostic tools would enable prompt isolation of OXA-48-producing CRE and, hence, provide targeted treatment while treating the infection.

**Figure 11:**
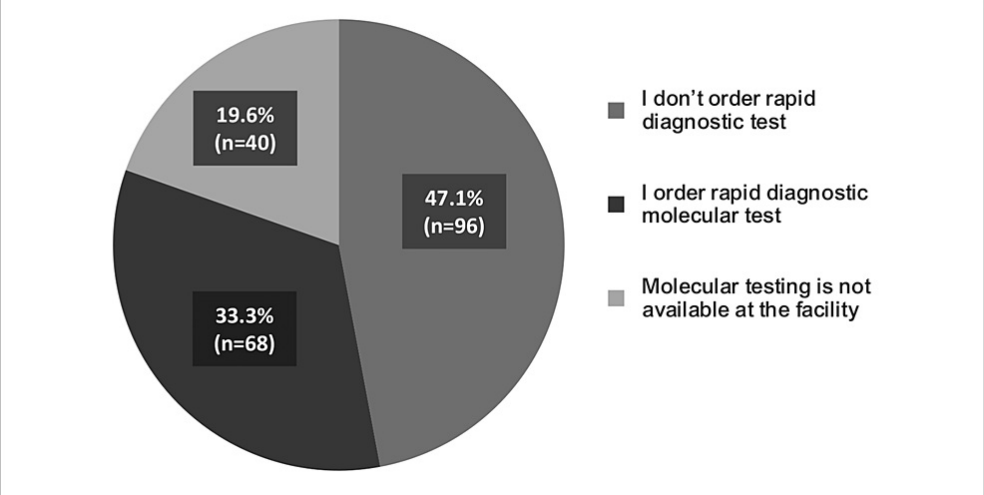
To detect a common molecular mechanism of carbapenem resistance in Enterobacterales like OXA-48, and NDM in my healthcare setting.

**Figure 12:**
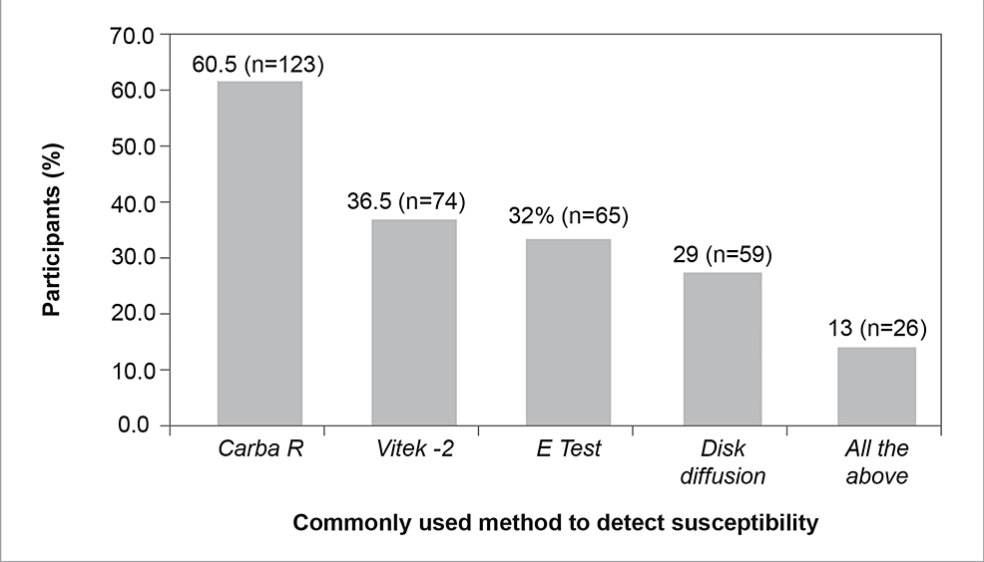
Commonly used methods to detect the susceptibility of CAZ-AVI in my hospital setting (Select multiple options, if applicable)

**Figure 13:**
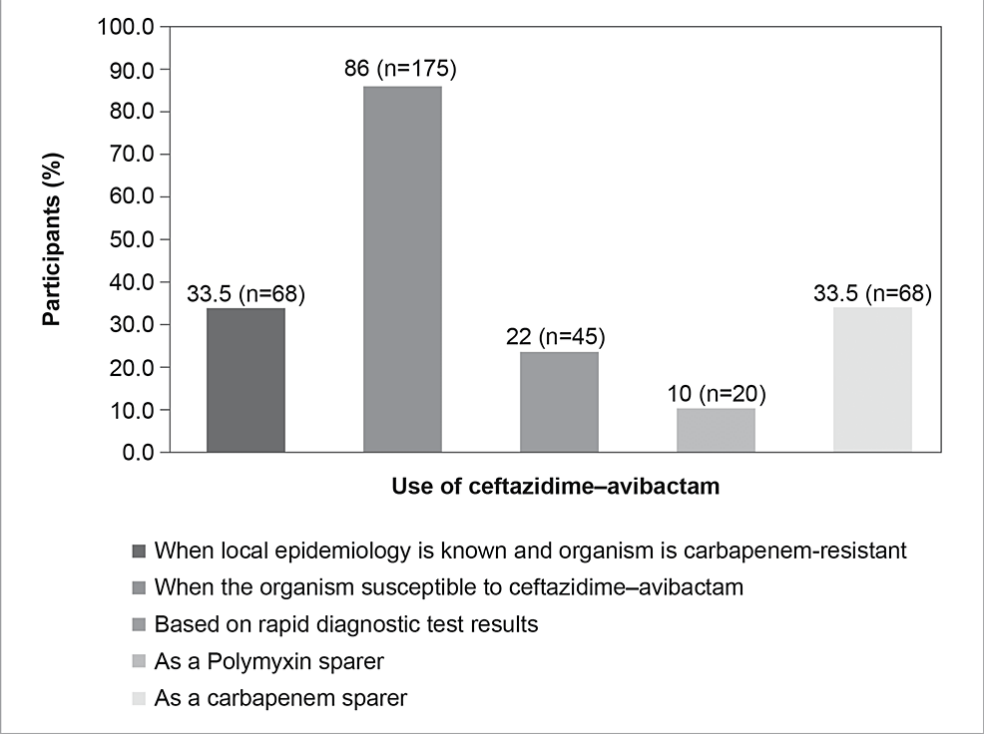
I use CAZ-AVI in the following while treating MDR gram-negative infection (Select multiple options, if applicable)

**Figure 14:**
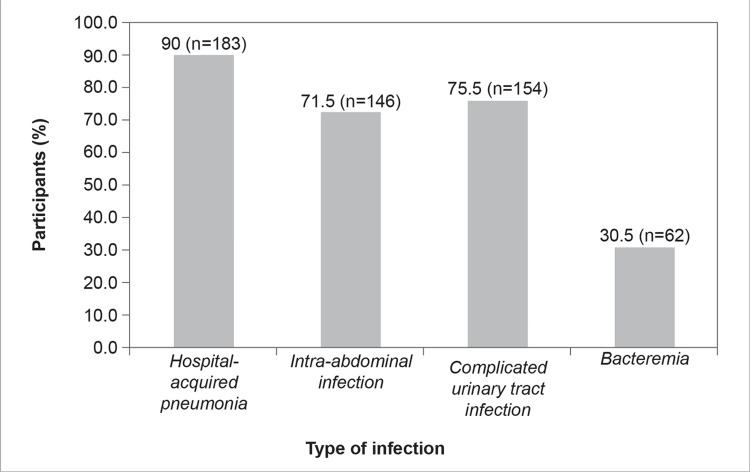
CAZ-AVI is considered a treatment option for the following infections in the hospital setting (Select multiple options, if applicable)

**Figure 15:**
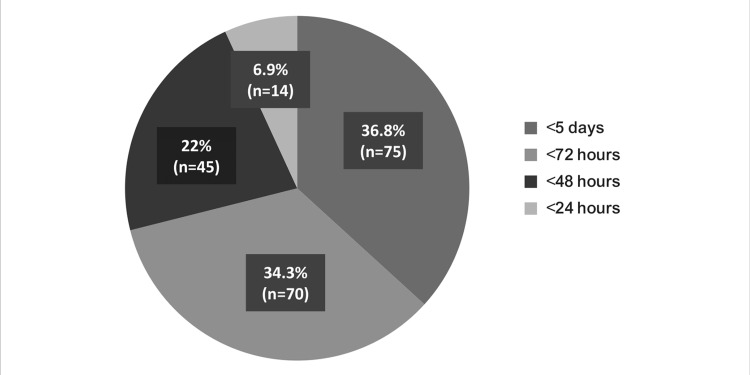
How early is CAZ-AVI used for the management of MDR gram-negative infections in your hospital setting?

**Figure 16:**
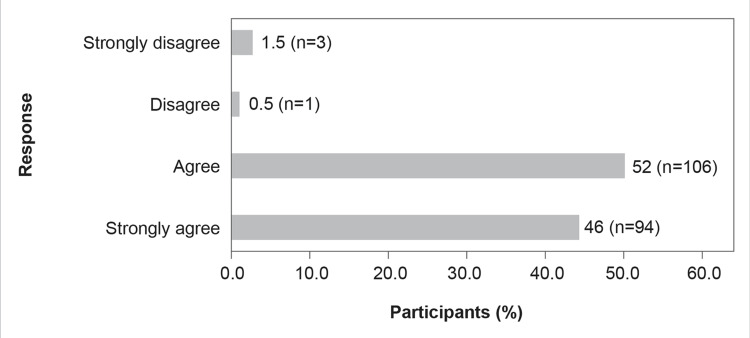
In my experience, early and appropriate use of CAZ-AVI leads to better patient outcomes when susceptible

Responses of HCPs to the KAP survey on questions regarding the use of CAZ-AVI in Indian hospital settings are illustrated in Figures 11-16. Nearly 33.3% (n=68) of participants opined that they order rapid diagnostic molecular tests, whereas 19.6% (n=40) did not have a molecular testing facility in their settings (Figure 11).

Most of the participants used Carba R (n=123, 60.5%), followed by Vitek®-2 (n=74, 36.5%), E test (n=65, 32%), and disk diffusion (n=59, 29%) to detect the susceptibility of CAZ-AVI or to detect the molecular mechanism of carbapenem resistance in their hospital settings, whereas 13% (n=26) of the participants used all the mentioned tests (Figure 12).

Almost 86% (n=175) of participants preferred using CAZ-AVI when the organism was susceptible to it. Other factors governing the choice of CAZ-AVI were included when the local epidemiology was known and the organism was carbapenem-resistant (n=68, 33.5%) or based on rapid diagnostic test results (n=45, 22%). Around 10% (n=20) of the respondents opined to use CAZ-AVI as a polymyxin sparer and 35.5% (n=68) as a carbapenem sparer (Figure 13).

Most of the participants (n=183, 90%) opined that CAZ-AVI is a therapeutic option for hospital-acquired pneumonia, with urinary tract infections being the second most frequent indication (75.5%, n=154) (Figure 14).

Finally, almost 98% (n=200) of the participants agreed that early and appropriate use of CAZ-AVI leads to better patient outcomes when the pathogen is susceptible. Furthermore, the question regarding the time to initiate CAZ-AVI therapy had the following responses: 7% (n=14) opined within 24 hours, 22% (n=45) opined within 48 hours, 34% (n=70) opined within 72 hours, and 37% (n=75) opined within five days (Figures 15-16).

## Discussion

Treatment options for common infections have become limited due to the development and escalation of resistant bacteria with new resistance mechanisms [1,2]. Due to their lipopolysaccharide-containing complex cell envelope, which imparts it a distinct structure, gram-negative bacteria are more resistant than gram-positive bacteria and, hence, are a major source of IDs and mortality globally. The WHO has identified MDR gram-negative pathogens, such as CRE, *A. baumannii*, and *P. aeruginosa*, as the highest priority for the development of newer antimicrobials [1,16].

As per the Indian Council of Medical Research (ICMR) AMR annual report, over the last six years, *K. pneumoniae* prevalence decreased consistently from 65% in 2016 to 43% in 2021, whereas the overall prevalence of carbapenem susceptibility of *E. coli* dropped slowly from 86% in 2016 to 64% in 2021 [17].

The results of the present survey show that *K. pneumoniae*, followed by *E. coli*, *P. aeruginosa*, and *A. baumannii*, remain the most common gram-negative nosocomial pathogens encountered in Indian ICUs (Figure 3); however, as per the ICMR AMR annual report, *E. coli* was the most commonly isolated pathogen, followed by the *K. pneumoniae*, *A. baumannii*, *P. aeruginosa*, and S*taphylococcus aureus* [17].

The present survey revealed that hospital-acquired pneumonia was the most encountered infection, followed by sepsis, urinary tract infection, and intra-abdominal infection (Figure 4). A similar study conducted by Dasgupta et al. (2015) in a tertiary care center in India revealed that pneumonia, ventilator-associated pneumonia, urinary tract infection, and bloodstream infections were the most common nosocomial infections in the clinical setting [18]. Studies have reported that the respiratory system, urinary system, and blood are the most common locations of nosocomial infections in ICUs [19].

Newer antibiotics under development provide therapeutic benefits over current therapies, and drug resistance to these novel antibiotics is anticipated to emerge rapidly within months or years [20,21]. AMR is being further exacerbated by the ongoing failure to develop and manufacture newer antibiotics, which jeopardizes our capacity to effectively treat bacterial infections. Hence, the inability to produce new antibiotics emphasizes the requirement to choose optimal methods for treating bacterial infections [20,21]. The present KAP survey results show that most Indian HCPs do not believe that there is a global shortage of innovative antibiotics, and it is contributing to the rising incidence of antibiotic resistance (Figure 1). Most of the HCPs participated in the KAP survey also voted that new antibiotics would add to the armamentarium of treatment options for MDR gram-negative infections as antibiotic development pipelines run dry (Figure 5). Hence, a consolidated effort of implementing effective AMS, along with optimizing the utility of antimicrobial agents and renewed research endeavors, is the need of the hour to substantially manage this AMR crisis [3]. A meta-analysis of 22 studies conducted by Martin et al. (2018) showed that serious infections caused by CRE in patients who are hospitalized have a higher fatality rate than infections caused by carbapenem-sensitive *Enterobacterales*. The study also showed that compared to carbapenem-sensitive *K. pneumoniae*, carbapenem-resistant *K. pneumoniae* had a substantially increased risk of overall death [22]. At present, very limited options for the treatment of CRE infections are accessible, which further highlights the need for innovative antibiotics [23].

The results of the survey reflect that 26.5% of HCPs agree that CAZ-AVI can be the primary choice of therapy for infections caused by OXA-48-producing CRE (Figure 8). A study by Temkin et al. (2016) showed that, whether taken alone or in combination with other antibiotics, CAZ-AVI successfully treated infections caused by carbapenem-resistant microorganisms (95% of which had previously failed treatment) [24]. The survey results also show that while most HCPs agree that CAZ-AVI is an important tool in their armamentarium to tackle MDR gram-negative infections, AMS needs to be practiced for the use of CAZ-AVI (Figures 6-9). ICMR has recommended that CAZ-AVI can be used as a first-line treatment option against OXA-48-like CRE [25]. The findings of the Antimicrobial Testing Leadership and Surveillance study, which tested the in vitro activity of CAZ-AVI and its comparators in *E. coli* and *K. pneumoniae* isolated from different tertiary centers in India, showed that CAZ-AVI is a potential therapeutic option for CRE, including OXA-48-producing *K. pneumoniae* and *E. coli* [26]. As a monotherapy, CAZ-AVI shows promising results in treating patients with severe infections caused by OXA-48-producing *Enterobacterales* and who have few alternative treatment options [27].

As per the survey, where the participants opined that early initiation of antibiotic treatment can improve clinical outcomes (Figure 16), around 34% of the participants recommended <72 hours and 22% recommended <48 hours for using CAZ-AVI for the treatment of gram-negative infections caused by MDR bacteria in their hospital settings (Figure 15). However, it was observed that almost half of the participants do not order a rapid diagnostic test, whereas nearly 20% do not have a molecular testing facility at their centers (Figure 11). This might pose a significant challenge in the use of CAZ-AVI because rapid detection of gram-negative bacteria that produce carbapenemase is essential for therapy optimization and prevention of the further spread of these organisms.

As discussed above, the importance of early initiation of antibiotic therapy was highlighted in the results obtained by Jorgensen et al. (2019). Early initiation of CAZ-AVI (within 48 hours of the onset of infection) was observed to improve clinical outcomes [28]. This was also highlighted in several other studies, which showed that an interruption in the initiation of antibiotic therapy can lead to serious negative consequences [29-33]. Therefore, rapid diagnosis, identification of pathogens, and susceptibility testing are immensely important in strategizing management protocols and antibiotic use [28,29]. An observational study conducted by Satlin et al. (2022) explained that β-lactamases KPC PCR testing was associated with a shorter time to receipt of active therapy and decreased 30-day mortality, and initial CAZ-AVI-targeted treatment was linked to lesser 30-day mortality than polymyxin [34].

Newly developed molecular diagnostic methods like multiplex PCR can yield results within one to four hours, which can lead to improved clinical outcomes, decreased mortality, and reduced hospitalization duration and costs [28,35-38]. Rapid diagnostic tools, coupled with AMS programs, can lead to an overall improvement in the implementation of management strategies and standardization of stewardship policies [28,39]. Most of the participants (90%, n=183) opined that CAZ-AVI is considered a treatment option for hospital-acquired pneumonia, with urinary tract infections being the second most recurrent indication (75.5%, n=154) (Figure 14). Results from several reports show that CAZ-AVI is most commonly considered a treatment option for hospital-acquired pneumonia followed by complicated urinary tract infections, intra-abdominal infections, and bacteremia [11,28]. Real-world evidence published by Jorgensen et al. showed that CAZ-AVI is presently employed to treat several MDR gram-negative infections, including *Pseudomonas* spp. and CRE [28]. Tumbarello et al. observed that 30-day mortality in patients who received CAZ-AVI was 36.5% compared to 55.8% in the control population [40]. These results are promising because most of the study patients had a wide range of difficult medical disorders and high-index sickness severity. Furthermore, these studies demonstrated that CAZ-AVI was effective, safe, and well-tolerated among patients [11,12,28,29,40].

In the KAP survey, most HCPs believed that they would prefer CAZ-AVI when local epidemiology is known and the organism is carbapenem-resistant (Figure 13). However, despite the rising incidence of resistance in India, there remains a paucity of data on the epidemiology and the country's antibiotic resistance mortality burden [41]. However, only around 10% of the participating HCPs use CAZ-AVI as a polymyxin sparer (Figure 13). Studies that evaluate the epidemiology and local susceptibility patterns are, hence, the need of the hour. The continued use of polymyxins in India is concerning, given its clinical inefficacy and nephrotoxicity. Recent studies that compare polymyxins with newer gram-negative agents show that polymyxins have a decreased clinical response and increased mortality [42-45].

The limitation of this study is that the KAP survey has included only a small number of participants. We propose that the results should be confirmed with a larger cohort in the future.

## Conclusions

The present KAP survey was used to understand the gaps between evidence and clinical practice. The outcomes of the survey conclude that CAZ-AVI can be a potential alternative for the treatment of MDR gram-negative bacterial infections, specifically those that have developed resistance to most of the currently used drugs. HCPs are facing challenges in introducing novel antimicrobial drugs into clinical practice, owing to the need for appropriate AMS programs and gaps in the widespread availability of rapid diagnostic tests. The HCPs will be able to use these agents more effectively in routine clinical practice by comprehending and overcoming these difficulties. However, more rigorous AMS is required in the process, which reiterates the need for increased awareness and rapid diagnostic methods.
